# Supplementary material for: Effect of Particle Size of Silage of Flint Corn Grain on Dairy Cows Fed Tropical Pasture: Performance, Intake, Ruminal Fermentation, and Digestibility
Source: Animals (Basel). 2023 Jun 9;13(12):1932. doi: 10.3390/ani13121932 (PMC10295517; doi:10.3390/ani13121932)
Supplement: Supplementary file 1 [file animals-13-01932-s001.zip › animals-2254047-supplementary.pdf]

animals

MDPI

Supplementary Figures: Effect of Particle Size of Silage of Flint Corn Grain on Dairy Cows Fed Tropical Pasture: Performance, Intake, Ruminal Fermentation, and Digestibility

Débora R. Gomide [1,2], Renata A. N. Pereira [2], Rayana B. Silva [3], Josué T. R. Carvalho [1], Márcio A. S. Lara [1] and Marcos N. Pereira [1,*]

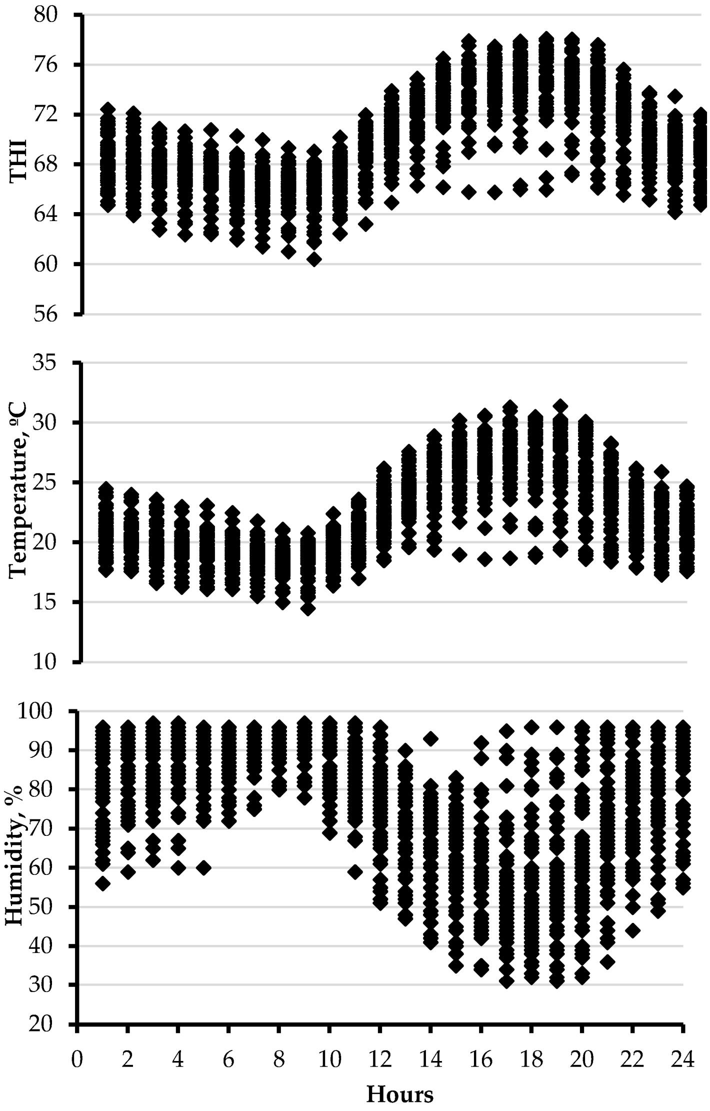

Figure S1. Temperature–humidity index (THI), temperature, and humidity during the experiment. 1483 recordings at 1 h intervals. Temperature: 22.3 ± 3.6 °C (mean ± SD). Humidity: 76.0 ± 7.8%. THI: 69.8 ± 3.6.

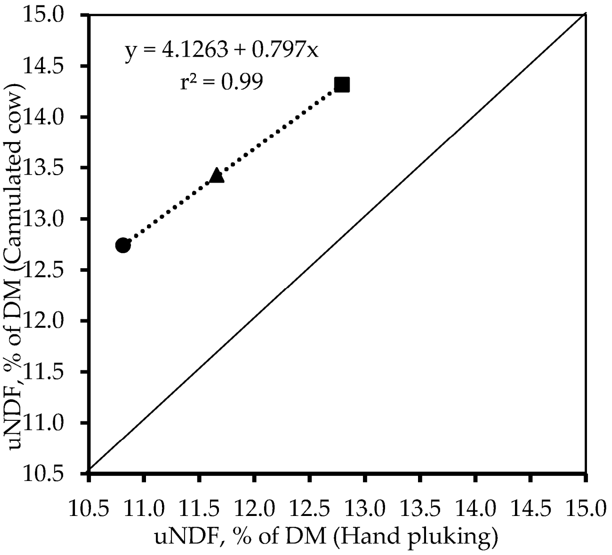

(a)

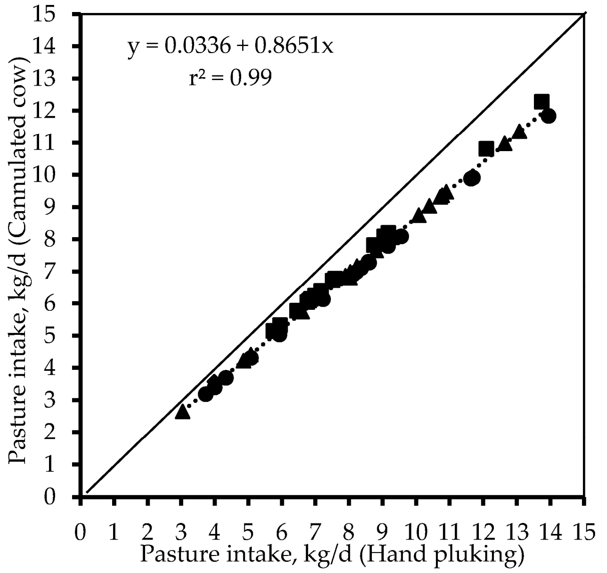

(b)

Figure S2. (**a**) Undigested neutral detergent fiber (uNDF) of pasture samples collected by hand plucking or by a cow with evacuated rumen; (**b**) pasture intake estimated with uNDF analyzed with pasture samples collected by hand plucking or by a cow with evacuated rumen (■ period 1; ▲ period 2; ● period 3).
